# Identification and Prioritization of Canadian Society of Nephrology Clinical Practice Guideline Topics With Multidisciplinary Stakeholders and People Living With Kidney Disease: A Clinical Research Protocol

**DOI:** 10.1177/20543581231207142

**Published:** 2023-11-24

**Authors:** Brigitte H. Baragar, Melissa Schorr, Nancy Verdin, Tania Woodlock, David A. Clark, Gregory L. Hundemer, Anna Mathew, Reem A. Mustafa, Krista S. Ryz, Tyrone G. Harrison

**Affiliations:** 1 Department of Medicine, University of Manitoba, Winnipeg, Canada; 2 Department of Medicine, Western University, London, ON, Canada; 3 Department of Health Research Methods, Evidence, and Impact, McMaster University, Hamilton, ON, Canada; 4 Patient and Community Engagement Research Unit, O’Brien Institute for Public Health, University of Calgary, AB, Canada; 5 Division of Nephrology, Department of Medicine, Dalhousie University, Halifax, NS, Canada; 6 Kidney Research Institute Nova Scotia, Queen Elizabeth II Health Sciences Centre, Halifax, NS, Canada; 7 Ottawa Hospital Research Institute, University of Ottawa, ON, Canada; 8 Division of Nephrology, Department of Medicine, University of Ottawa, ON, Canada; 9 Division of Nephrology, Department of Medicine, McMaster University, Hamilton, ON, Canada; 10 Departments of Internal Medicine and Population Health, The University of Kansas Health System, Kansas City, USA; 11 Department of Medicine, University of Calgary, AB, Canada; 12 Department of Community Health Sciences, University of Calgary, AB, Canada; 13 O’Brien Institute for Public Health, Cumming School of Medicine, University of Calgary, AB, Canada; 14 Libin Cardiovascular Institute, Cumming School of Medicine, University of Calgary, AB, Canada

**Keywords:** clinical practice guideline, kidney disease, patient engagement, priority setting, topic prioritization

## Abstract

**Background:**

Despite efforts to provide evidence-based care for people living with kidney disease, health care provider goals and priorities are often misaligned with those of individuals with lived experience of disease. Coupled with competing interests of time, resources, and an abundance of suitable guideline topics, identifying and prioritizing areas of focus for the Canadian nephrology community with a patient-oriented perspective is necessary and important. Similar priority-setting exercises have been undertaken to establish research priorities for kidney disease and to standardize outcomes for kidney disease research and clinical care; however, research priorities are distinct from priorities for guideline development. Inclusion of people living with health conditions in the selection and prioritization of guideline topics is suggested by patient engagement frameworks, though the process to operationalizing this is variable. We propose that the Canadian Society of Nephrology Clinical Practice Guideline Committee (CSN CPGC) takes the opportunity at this juncture to incorporate evidence-based prioritization exercises with involvement of people living with kidney disease and their caregivers to inform future guideline activities. In this protocol, we describe our planned research methods to address this.

**Objective:**

To establish consensus-based guideline topic priorities for the CSN CPGC using a modified Delphi survey with involvement of multidisciplinary stakeholders, including people living with kidney disease and their caregivers.

**Study design:**

Protocol for a Modified Delphi Survey.

**Setting:**

Pilot-tested surveys will be distributed via email and conducted using the online platform SurveyMonkey, in both French and English.

**Participants:**

We will establish a group of multidisciplinary clinical and research stakeholders (both within and outside CSN membership) from Canada, in addition to people living with kidney disease and/or their caregivers.

**Methods:**

A comprehensive literature search will be conducted to generate an initial list of guideline topics, which will be organized into three main categories: (1) International nephrology-focused guidelines that may require Canadian commentary, (2) Non-nephrology specific guidelines from Canada that may require CSN commentary, and (3) Novel topics for guideline development. Participants will engage in a multi-round Modified Delphi Survey to prioritize a set of “important guideline topics.”

**Measures:**

Consensus will be reached for an item based on both median score on the Likert-type scale (≥ 7) and the percentage agreement (≥ 75%); the Delphi process will be complete when consensus is reached on each item. Guideline topics will then be given a priority score calculated from the total Likert ratings across participants, adjusted for the number of participants.

**Limitations:**

Potential limitations include participant response rates and compliance to survey completion.

**Conclusions:**

We propose to incorporate evidence-based prioritization exercises with the engagement of people living with kidney disease and their caregivers to establish consensus-based guideline topics and inform future guidelines activities of the CSN CPGC.

## Abrégé

**Contexte:**

Malgré les efforts déployés pour fournir aux personnes atteintes de néphropathies des soins fondés sur les données probantes, il s’avère que les objectifs et priorités des prestataires de soins de santé sont souvent mal alignés avec ceux des personnes qui ont une expérience concrète de la maladie. Compte tenu des intérêts concurrents en matière de temps et de ressources, et de l’abondance de sujets pertinents pour l’élaboration de lignes directrices, il est nécessaire et important d’identifier et de hiérarchiser les domaines prioritaires pour la communauté néphrologique canadienne dans une perspective orientée vers le patient. Des exercices semblables pour la définition des priorités ont été entrepris afin d’établir les priorités de la recherche sur les maladies rénales et normaliser les soins cliniques et les résultats de la recherche sur les maladies rénales. Or, les priorités de la recherche diffèrent des priorités pour l’élaboration des lignes directrices. Bien que les façons de procéder varient, les cadres d’engagement des patients suggèrent que des personnes vivant avec des problèmes de santé soient incluses dans la sélection et la hiérarchisation des sujets des lignes directrices. À cet égard, nous proposons que le Comité sur les lignes directrices de pratique clinique de la Société canadienne de néphrologie (CSN CPGC — Canadian Society of Nephrology Clinical Practice Guideline Committee) profite de l’occasion pour intégrer des exercices d’établissement des priorités fondés sur des données probantes et impliquer des personnes vivant avec une néphropathie et des soignants afin de guider les futures activités d’élaboration de lignes directrices. Dans ce protocole, nous décrivons la méthodologie de recherche que nous suivrons pour y remédier.

**Objectif:**

Établir des priorités consensuelles en matière de sujets de lignes directrices pour le CSN CPGC à l’aide d’une enquête Delphi modifiée et avec la participation d’intervenants multidisciplinaires, notamment des personnes vivant avec une néphropathie et leurs soignants.

**Conception:**

Protocole pour une enquête Delphi modifiée.

**Cadre:**

Des sondages pilotes, en anglais et en français, seront distribués par courriel et réalisés à l’aide de la plateforme en ligne SurveyMonkey.

**Participants:**

Nous créerons un groupe d’intervenants multidisciplinaires canadiens œuvrant en clinique et en recherche (à la fois des membres et des non membres de la SCN) auquel s’ajouteront des personnes atteintes d’une néphropathie et/ou leurs soignants.

**Méthodologie:**

Une recherche exhaustive sera effectuée dans la littérature afin de constituer une première liste de sujets de lignes directrices, laquelle sera divisée en trois catégories principales: (1) lignes directrices internationales axées sur la néphrologie et pouvant nécessiter des commentaires canadiens, (2) lignes directrices canadiennes non spécifiquement liées à la néphrologie et pouvant nécessiter des commentaires de la SCN, (3) nouveaux sujets pour l’élaboration de lignes directrices. Les participants s’engageront dans une enquête Delphi modifiée à plusieurs tours afin de hiérarchiser un ensemble de « sujets importants pour l’élaboration de lignes directrices ».

**Mesures:**

Un consensus sera atteint pour un énoncé s’il atteint à la fois un score médian (≥7) sur l’échelle de Likert et le pourcentage d’accord établi (≥ 75 %); le processus Delphi sera terminé lorsque le consensus sera atteint pour chaque énoncé. Les sujets pour l’élaboration de lignes directrices recevront ensuite une cote de priorité calculée à partir du total des scores des participants sur l’échelle Likert et ajustée en fonction du nombre de participants.

**Limites:**

L’étude pourrait être limitée par le taux de réponse des participants et leur engagement à compléter toutes les étapes de l’enquête.

**Conclusion:**

Nous proposons d’intégrer des exercices de définition des priorités fondés sur des données probantes et impliquant la participation de personnes vivant avec une néphropathie et de leurs soignants afin de déterminer des sujets consensuels pour l’élaboration de lignes directrices et de guider les futures activités du CSN CPGC en lien avec ce processus.

## Introduction

The global prevalence of kidney disease is increasing, with a prevalence in Canada reported to be 12.5%.^1^ Over 6000 people in Canada develop kidney failure each year and it is the eleventh leading cause of death.^2^ People living with chronic kidney disease face high symptom burden and are among the most complex groups in the medical community.^3,4^ Many challenges exist in managing the coexisting comorbidities, symptoms, and day-to-day demands of people with living kidney disease.^3^ Ongoing medical research continues to discover innovative diagnostic and therapeutic strategies and, thus, clinical practice guidelines require frequent review and adaptation.^5^ Coupled with competing interests of time, resources, and an abundance of possible guideline topics, identifying and prioritizing areas of focus for guideline development and commentary in the Canadian kidney community is necessary and important.

Guideline standards have highlighted the importance of engaging diverse stakeholders in guideline development, including people living with health conditions and their caregivers, to reduce the potential for bias in topic selection.^6,7^ Furthermore, diverse participation of stakeholders can ensure guideline topics are relevant to both those who are delivering care and those receiving care, and thus directly impacted by guideline implementation.^6^ Importantly, it has been shown that the goals and priorities of clinicians are often misaligned with those of individuals with lived experience of disease.^8^ For example, a recent study from Canada demonstrated that the involvement of people with chronic disease in population-level health care improvement decisions led to a meaningful shift in care priorities compared with similar committees that involved health care professionals alone.^9^

Priority-setting exercises that have included perspectives of people with lived experience of disease have been undertaken in the research community to establish research priorities for kidney disease^10-12^ and to standardize outcomes for kidney disease research and clinical care.^12-15^ Though related, research priorities are distinct from guideline topics and the engagement of people living with chronic disease in guideline topic prioritization remain relatively uncommon. Furthermore, how this engagement is operationalized in guideline organization recommendations is variable.^6,16^

The Delphi technique is a commonly used method for engaging stakeholders in both guideline development and in health research prioritization work.^16,17^ The Delphi technique is well suited for engaging diverse stakeholders given its flexible platform, multiple feedback processes, and preservation of participant anonymity.^17^ The modified Delphi technique is similar to its predecessor; however, instead of initial identification of topics of interest by a panel of participants, the process begins with a structured literature review. This adds more scientific rigor to the list of topics and brings focus for participants. Further modifications made to the traditional Delphi technique in this study include the use of online surveys rather than in person interviews and expanding the definition of stakeholders to include those living with disease and their caregivers.^18^

In this research protocol, we describe the planned work by the Canadian Society of Nephrology Clinical Practice Guideline Committee (CSN CPGC) to incorporate evidence-based prioritization exercises with involvement of people living with kidney disease, caregivers, as well as clinicians and researchers to prioritize guideline topics and inform future CSN CPGC activities.

## Methods

**Figure 1.**
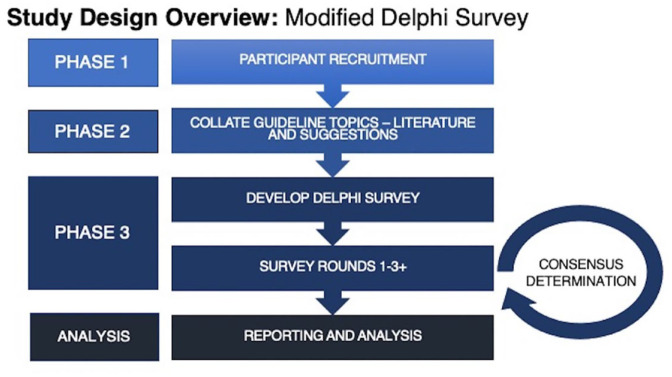
Study design overview including 4 phases: (1) participant recruitment; (2) collate guideline topics through literature review and suggestions from previous Canadian Society of Nephrology Clinical Practice Guideline Committee (CSN CPGC) activities; (3) develop and conduct Delphi survey with multiple rounds to target consensus determination; (4) analysis of results.

In this protocol, we describe our plans to identify and prioritize future guideline topics for the CSN CPGC with multidisciplinary stakeholder and patient involvement. We will conduct this study using the modified Delphi survey approach, which is outlined below and in Figure 1. Ethics approval for this study has been obtained through McMaster University (HiREB 14626).

### Participants and Recruitment

Participants on our Delphi panel will be comprised of a multidisciplinary group of Canadian health care professionals with clinical or research expertise in caring for those with kidney disease. This will include (but will not be limited to) nurses, physicians, pharmacists, dietitians, occupational and physical therapists, in addition to kidney disease researchers. People living with kidney disease and their caregivers will also be invited to participate through engaging with national and jurisdictional patient stakeholder groups and nephrology/research organizations, including the following:

1. Kidney Foundation of Canada (KFOC)
2. CanSolve CKD
3. Kidneylink.ca
4. Institute for Clinical Evaluative Sciences—Kidney Dialysis and Transplantation (ICES-KDT)
5. Provincial kidney organization patient forums (Patient and Family Area Councils as part of the Ontario Renal Network, Alberta Kidney Care, British Columbia Renal Agency etc.)
6. Canadian Association of Pediatric Nephrologists (CAPN)
7. Canadian Society of Transplantation (CST)
8. Canadian Society of Nephrology (CSN)
9. Société québécoise de néphrologie (SQN)

Delphi panels are developed using non-probability sampling, with purposive selection of participants.^19^ This means that we will rely on our collective experience and judgment to recruit appropriate participants. There is no standard sample size for Delphi methods, though a recent systematic review of Delphi methods used in health care identified 80 studies with a median number of participants of 17 (interquartile range [IQR] = 20).^20^ Larger Delphi panels are sometimes used depending on study aims and desired variety of expertise, though this can lead to survey fatigue since more Delphi rounds are generally needed to satisfy consensus criteria.^21^ However, if the panel is purposively heterogeneous, a larger participant number is necessary to enhance the validity of the results.^19^ We will aim to recruit 20 people living with kidney disease or caregivers, in addition to the anticipated larger number from the clinical, research, and nephrology organizational groups. From those that agree to participate, we predict that we will have approximately 40% to 70% of participants that will complete all surveys throughout the Delphi rounds.^21,22^ With this in mind, we aim to recruit an estimated 130 participants to be involved in initial rounds of the Delphi process. We will attempt to achieve diversity in sex/gender, career stage, location, and profession in our sampling of multidisciplinary participants. With the anticipated challenges of recruiting people with lived experience, we will not be as selective in terms of diversity of patient recruitment.

We will ask invited participants to suggest additional members for inclusion as per the criteria listed above (ie, snowball sampling, another non-probability-based sampling method). This will enhance our recruitment of panelists with more of a clinical focus and those outside of the CSN membership. If our initial recruitment efforts do not meet our intended sample size requirements, invitations to participate will also be shared publicly with the Canadian kidney community through social media platforms, such as Twitter and Facebook.

### Initial Survey—Demographics and Suggestions From Panelists

Surveys will be designed using the online platform SurveyMonkey, in both French and English.^23^ For our participants with living with kidney disease and their caregivers, the initial survey will include demographics, such as sex at birth, gender identity, age, cultural background, province of residence, awareness of guidelines, and perceived importance of involving people with lived experience. For clinical or research participants, we will ask the number of years practicing, primary professional role, secondary professional role, expertise in kidney medicine, and expertise in guideline development and use. We will also ask participants for suggestions of guideline topics, which will be incorporated into future prioritization rounds of the Delphi survey method.

This initial survey will be piloted for face and content validity among committee members and 1 to 3 additional people external to the project design. The survey will be piloted to assess the usability of the SurveyMonkey interface, the clarity of the items, and length of the survey; this will inform modifications necessary before dissemination.

Participants will be given 4 weeks to complete the survey, with weekly reminders sent by email to non-responders for the 4 weeks following the initial survey email. Non-respondents will not be propagated in further rounds.

### Delphi Ranking Survey Rounds

The Canadian Society of Nephrology Clinical Practice Guideline Committee members will develop an initial list of topics based on literature review and discussion, which will be included in the first ranking Delphi survey round. Our focused literature review will systematically search Medline to identify: (1) International kidney guidelines that may warrant Canadian contextualization via commentary from the CSN CPGC, (2) Canadian guidelines from non-nephrology guideline organizations where care of other disease states may differ in patients living with kidney disease compared with the general population, and (3) Novel guideline topics. Novel guideline topics are those that have not yet been the focus of a nephrology guideline body (either the CSN or internationally); this may be a guideline about kidney specific conditions or related to management of a non-kidney condition in people with kidney disease, among others. The search strategy will include multiple medical subject title terms and keywords relating to kidney disease and guidelines. The search will be limited to human and English articles published within the last 3 years. Topics suggested in previous CSN CPGC activities will be included as well and the literature review derived list of guideline topics will be supplemented by suggestions from the initial survey. All topics will be reviewed by CSN CPGC members for feasibility and care will be taken to ensure that the topics of people with lived experience of kidney disease and caregivers are clarified for feasibility considerations to ensure they are propagated into the Delphi survey process.

In the first Delphi prioritization round, feasible recommendations will be grouped into the 3 major categories of CSN CPGC activities listed above. For each potential guideline topic, a 9-point Likert-type scale will be included from Strongly Disagree (1) to Strongly Agree (9) that the topic is important. This is the most commonly used scale in Delphi surveys.^20,21^ Consensus will be reached for an item based on both median score (≥ 7) on the Likert-type scale *and* the percentage agreement (≥ 75%). The percentage agreement is determined by calculating the proportion of participants that ranked the item as ≥ 7 or < 7. If 80% of the participants ranked a topic as 7 or above, it would have 80% agreement (and meet consensus per our *a priori* determined criteria). In addition, if a median rating for an item was less than 7, with at least 75% percentage agreement, it will not be included in the subsequent surveys. If an item had a median rating ≥ 7 but did not meet percentage agreement criteria, it will be propagated to the next survey round. We will administer additional ranking surveys until percentage agreement is reached (anticipating 2-3 ranking survey rounds). The second (and subsequent) ranking survey(s) will include the group median ranking and percentage agreement with each topic item, so that, participants calibrate their ratings based on where the group consensus currently sits. Once all the items have reached consensus, the Delphi process is completed, and topics will be ranked by determining a mean priority score. This will involve adding the Likert score for each topic across individuals in the round that consensus was achieved for that specific topic. We will be able to prioritize all topics this way, and additionally stratified into the 3 categories of topics.

### Reporting and Analysis of Delphi Survey

Participant demographics will be summarized in a table. A summary figure showing the participation (and attrition) between rounds, in addition to number of items propagated between rounds, will be created as recommended in the literature.^20,21^ A summary table outlining the items that met consensus criteria for recommendation will be created, with median ranking (with IQR), percentage agreement, and round in which consensus was reached being included. A full list of the items that were ranked will be drafted as an additional (supplementary) table, including where the source of the recommendation was (panelist or literature). We will stratify and compare priorities between stakeholder groups descriptively. If priorities are significantly different, we may consider inverse weighting, so that, stakeholder groups with less representation are not disadvantaged by aggregating responses with the larger group.

## Discussion

This protocol summarizes our approach to a collaborative prioritization of guideline topics in kidney disease for the CSN CPGC to focus on. Our proposed work has several strengths. The engagement of people with lived experience of kidney disease, their caregivers, and multidisciplinary stakeholders to direct future guideline activities of the CSN is novel, important, and a priority for the CSN and the Canadian kidney community overall. Our proposed work will allow the CSN CPGC to ensure our guidelines are patient-oriented from the outset of guideline development, which will enhance Canadian clinicians’ provision of person-centered care. Enabling those living with disease states to take an active role has recognized benefits, including improved health care quality, improved cost-effectiveness of care, and improved individual, caregiver, and staff satisfaction.^24^

Our study proposes to recruit people with lived experience of kidney disease and their caregivers by including national patient stakeholder groups in our recruitment. Though members of patient stakeholder groups are only a subset of the Canadian population living with kidney disease, our recruitment strategy will be purposively broad to encourage diversity and heterogeneity in those participating. By recruiting through a variety of patient stakeholder groups we aim to represent the broad and varied perspectives of the Canadian kidney community across the kidney disease spectrum and lifespan. Furthermore, those engaged with stakeholder groups may be more likely to be interested in study participation and are a feasible group for recruitment with potentially lower risk of study attrition. The format of our survey delivery is easily accessible through its user-friendly virtual format and we anticipate this will increase study participation nationwide.

We acknowledge there are several limitations of our planned study. We have chosen to use a modified Delphi survey for this study, though there are other methods that can be utilized for priority setting. Another common method would be a James Lind Alliance (JLA) Priority Setting Partnership (PSP).^17^ Though the JLA-PSP dialogue is richer and may allow for qualitative data to be collected to inform prioritization, it is less feasible due to the cost of in person meetings in a geographically diverse country, such as Canada, challenges of the ongoing COVID-19 pandemic, and time. In addition, this study will rely on the participation of those recruited as well as participant survey completion, which may be difficult given that the completion is electronic and reminders will also be sent electronically. We have planned for this by ensuring that we have a thorough recruitment strategy with relatively high estimated attrition. Furthermore, we recognize that certain guideline topics may include medical terminology that is not widely understood by participants who have not received medical training. We will aim to minimize medical jargon as much as possible in guideline topics and provide opportunity for questions when topics are not clear. We have also engaged two patient and family member co-researchers (N.V. and T.W.), to ensure that our patient-facing documents include patient-friendly language and sufficient descriptions throughout. Of note, each survey round will have both a clinician/researcher version and a patient/family member/caregiver version to ensure that we are delivering targeted and appropriate surveys to both sets of stakeholders. Finally, in the Delphi ranking survey rounds, there will be transparency to allow for survey respondents to see median group rankings and percentage agreement. This will be helpful for participants to better understand differences in priority within the group, however, it could potentially bias the responses of participants on consensus surveys (ie, regression to the group median). Such bias would be difficult to quantify however, as response changes during consensus surveys are expected to achieve percentage agreement criteria. As this is an expected component of the Delphi methodology, we do not think that this is a significant limitation, but we should be aware of this when we eventually interpret survey results.

The prioritized guideline topics generated from our proposed study will serve as a master list of ideas to inform yearly CSN CPGC exercises to direct committee efforts, and we will aim to repeat guideline topic prioritization exercises every 5 to 10 years with the Canadian kidney community as feasible. The next steps of this work will be to determine a process within the CSN CPGC to assess the feasibility of providing commentary for each prioritized topic from the master list generated from this study. This is important to ensure the committee is drawing from this list of prioritized topics while still being guided by their own scope and limitations, as each priority still may be limited for feasibility reasons. Factors that may play a role in feasibility include the timeliness of the topic, the availability of well-done evidence synthesis, such as existing systematic reviews, variability in practice that needs evidence-based recommendations to inform/standardize, and the emergence of new practice changing evidence.

## Conclusion

It is critical to identify and understand differences in priorities between clinicians and researchers (who are often involved in guideline development) with people who have lived experience with kidney disease who are often excluded from guideline development though are most impacted by guideline recommendations. In this research protocol, we propose to establish consensus-based guideline topic priorities for the CSN CPGC using a modified Delphi survey with involvement of people living with kidney disease, their caregivers, and multidisciplinary stakeholders. Prioritized topics which emerge from this study will inform future clinical practice guideline activities within the CSN and broader Canadian kidney community.
